# Life after prostate cancer treatment: a mixed methods study of the experiences of men with sexual dysfunction and their partners

**DOI:** 10.1186/s12894-017-0231-5

**Published:** 2017-06-15

**Authors:** Jeffrey A. Albaugh, Nat Sufrin, Brittany R. Lapin, Jacqueline Petkewicz, Sandi Tenfelde

**Affiliations:** 1 John and Carol Walter Center for Urological Health, NorthShore University HealthSystem, 2180 Pfingsten Road, Suite 3000, Glenview, Illinois 60026 USA; 2 The Doctoral Program in Clinical Psychology, The City College of the City University of New York, New York, NY USA; 3 Cleveland Clinic, Cleveland, OH USA; 4 The Marcella Niehoff School of Nursing, Loyola University of Chicago, Maywood, IL USA

**Keywords:** Prostate cancer, Male cancers, Sexual dysfunction, Qualitative research, Phenomenology, Survivorship

## Abstract

**Background:**

Prostate cancer is the most common non-skin cancer in men and sexual dysfunction is the most frequently reported long-term side effect of prostate cancer surgery or radiation. The aim of this study was to examine the experiences of men with sexual dysfunction and their partners following prostate cancer treatment.

**Methods:**

Men with sexual dysfunction from either surgical removal or radiation therapy 1-5 years after treatment were interviewed, as well as their partners. A mixed method design was used to determine the lived experience of men with sexual dysfunction. Open-ended questions guided the interviews.

**Results:**

Twenty seven men completed the study with a mean age of 61 years (SD = 8.0; range = 44-77 years). Nine partners also participated. The majority of men (92.6%) had surgery. The average time from treatment to the interview was 23.5 months (SD = 11.7). Themes were frustration with sexual dysfunction, importance of support and understanding from others, depression and anxiety related to sexual dysfunction, importance of intimacy with partner, factors that impact treatment satisfaction, and education and comprehensive information about sex.

**Conclusions:**

Prostate cancer survivors and partners need accurate information about sexual side effects before during and after treatment. Men and partners required individualized help and guidance to manage sexual dysfunction. Support and understanding from partners, family, and others was also identified as an important aspect of healing and adjustment after prostate cancer treatment. Prostate cancer education/support groups played a key role in helping men and partners gain advocacy, education, and support. Psychological problems such as depression and anxiety need to be identified and addressed in men after prostate cancer treatment. Men and partners need assistance in understanding and navigating their way through intimacy to move forward with connectedness in their relationship. Satisfaction with treatment and with providers is dependent on patient education and understanding of all aspects of prostate cancer treatment including sexual side effects and incontinence.

## Background

One out of eight men will be diagnosed with prostate cancer during their lifetime, making the disease the most common non-skin cancer in the male population [1]. Although the majority of men will not die from prostate cancer, with survivorship as high as 93% for 15 years after diagnosis [1], treatment can have long-term side effects that greatly impact quality of life. Notably, sexual dysfunction is the most common side effect of both surgical removal of the prostate and radiation therapy [2]. While erectile dysfunction (ED) is not an immediate result of radiation therapy, a multicenter study has shown that sexual function in men who have undergone external beam radiation continues to decline to levels similar to men who had radical prostatectomy [3]. The incidence of ED has been reported as high as 79-88% after radical prostatectomy [4], despite advances in nerve sparing surgical techniques, and 67-72% after external beam radiation [5]. Helping men and their partners adjust to potential side effects such as sexual dysfunction following prostate cancer treatment is an important part of holistic care.

Erectile dysfunction impacts quality of life, with the majority of men reporting quality of life as either severely or moderately affected by ED [3, 6]. Several broad qualitative studies have been done to examine the lived experience of men with prostate cancer and have identified the challenges of sexual dysfunction after treatment in these men [7, 8], but little research was found looking at the lived experience of men specifically examining sexual dysfunction after prostate cancer treatment. Researchers examined the struggle towards the new normal with psychosexual adjustment after prostate cancer treatment in Australian men [7]. They found three main themes: Men were impacted by distressing sexual and urinary difficulties which negatively impacted self-perception and intimate relationships; receiving adequate information and support and good communication with providers and partners facilitated better adjustment; coming to terms with the side effects of prostate cancer treatment involved making lifestyle changes, coping and emotional struggles, while striving to accept/integrate the new normal self/life. Since an extensive Medline search did not reveal previous research to specifically examine the lived experience of men with sexual dysfunction after prostate cancer treatment and/or partners, the powerful descriptions from this study will provide new information on this phenomenon.

## Methods

### Sample/Study population

Participants were men who had undergone prostate cancer treatment within the last 1-5 years, with resulting sexual dysfunction. These men, and their partners, were asked to describe their experience with sexual dysfunction and prostate cancer treatment. Men and/or partners of men with prostate cancer were recruited through prostate cancer awareness/support/information groups or from the urology clinic of the principal investigator’s academic medical center. Recruitment was done through word of mouth and a recruitment flyer.

### Inclusion criteria

1. Men and/or partners of men with sexual dysfunction who underwent treatment for prostate cancer at least 1 year ago and no more than 5 years ago through either radical prostatectomy or radiation therapy.
2. Sexually active prior to and at the time of prostate cancer treatment.
3. Command of verbal and written English.

### Procedures

The IRB-approved study used a mixed methods design to determine the lived experience of men with sexual dysfunction and their partners. Participants individually, verbally answered open-ended questions about their treatment and experience. Interview sessions lasted as long as the participant needed (most lasting approximately 25 min) and were audio-recorded and transcribed. As is the norm with qualitative research, accrual stopped when the saturation level was achieved (when no new themes were emerging from the descriptions). In addition to open-ended interviews, participants, excluding partners, also completed quantitative questionnaires about erection hardness, erectile function and orgasm/climax quality.

### Analysis

Interview transcriptions were randomly spot checked for accuracy. Qualitative thematic text coding with a concentrated examination of the transcripts was performed using Dedoose [9, 10]. A code book was developed after two staff independently reviewed several transcripts to determine appropriate codes. The two coders met with a third coder to discuss and reconcile the codes in the code book. At this point, inter-rater reliability was assessed for all three coders, with a pooled Kappa value of 0.66. New codes were added by each of the three coders as they emerged, which were communicated to other coders. After initial coding was completed by the three independent coders, the coders met again to revisit and discuss all codes. The transcripts were re-assigned to different coders and further coding was done to increase inter-relater reliability, accuracy and comprehension. Following this phase, the group met to review codes and organize into emerging higher-order themes. All three reviewers submitted quotes from the interviews associated with the various themes. For the manuscript, the group reached consensus on 1-3 quotes for each theme that exemplified that particular theme. Quantitative data were analyzed using SAS version 9.3 (SAS Institute Inc., Cary, NC), which included calculation of descriptive statistics and measures of central tendency.

### Measures

Phenomenological Open Ended Questions: This qualitative study used open-ended questions to explore the experience of men with sexual dysfunction after prostate cancer treatment. Each man and/or partner was interviewed separately and asked an open-ended question: Please describe your journey with sexual dysfunction after prostate cancer treatment and/or how has sexual dysfunction impacted your life after prostate cancer treatment? Other guiding statements were used to help participants describe their lived experience such as: Is there anything else you want to tell us about your experience or that you think other people going through this or treating people going through this should know?

Erection Hardness Grading Score: Men were asked to grade the strength of their erections with the Erection Hardness Score, which is a validated measure. Erection strength was rated on a scale ranging from 0 to 4 with 0 (representing a 0% erection) being no erection at all and 4 (representing a fully hard 100% erection) being a completely hard erection [11].

The International Index of Erectile Function (IIEF) including the IIEF 5 and Overall Sexual Satisfaction: Sexual function was determined using the International Index of Erectile Function, which consists of a 15-item questionnaire measuring five domains including erectile function, orgasmic function, sexual desire, intercourse satisfaction, and overall sexual satisfaction, which were determined by principal components analysis [12]. Higher scores indicate higher functioning and satisfaction.

The Self-Esteem and Relationship Questionnaire (SEAR): Self-Esteem, sexual self confidence and participants’ relationship satisfaction were measured using the Self-Esteem and Relationship Questionnaire, which is a 14-item instrument developed as a patient-reported tool to assess sexual confidence and intimacy in a sexual relationship [13]. Higher scores indicate better satisfaction.

Orgasm/climax: In addition to questions used from the IIEF in the orgasm domain, orgasm/climax quality was evaluated by asking participants a single question to compare the quality of their orgasm to orgasms experienced prior to treatment. Men could respond to the question by circling a response of absent, diminished, normal or better as compared to prior to prostate cancer treatment.

Other Questions: The participants were asked demographic questions, if they had erectile dysfunction prior to prostate cancer treatment, if they had pain or leakage with orgasm, and about the type and length of time since prostate cancer treatment.

## Results

Twenty seven men completed the study, mean age 61 (±8) years (range 44-77 years). Nine partners of the men also participated in the interview process. Partners did not complete any forms. The large majority of men (93%) had surgical treatment. The average time from treatment to completion of study was 24 (±12) months (range 12-52 months). The majority of men did not report erectile dysfunction prior to prostate cancer (74%), however men reported post treatment erectile dysfunction with an average erection hardness of 1.48 (±1.2) (0-4 scale). The erectile dysfunction domain of the IIEF revealed a mean score of 16.1 (±5.8) out of a possible score of 30. All men except for two reported the ability to climax, but most men reported diminished orgasm quality (55.6%). Although the overall relationship satisfaction scores mean was high on the SEAR (76.9 ± 24.4), mean sexual relationship satisfaction was low (49.1 ± 26.3). For complete quantitative results including the results for IIEF and SEAR, see Table 1. Emergent themes from the qualitative interviews were the importance of education/comprehensive information about sex and/or sexual dysfunction, frustration with sexual dysfunction, the importance of support and understanding from others, the importance of intimacy in a relationship, the psychological ramifications of sexual dysfunction, and prostate cancer treatment provider satisfaction and/or dissatisfaction.

**Table 1:** Sample characteristics

Characteristics	N (%)
Total # of participants	27
Age (years), mean ± SD (range)	60.9 ± 8.0 (44-77)
Race	
Caucasian	22 (81.5)
Asian	1 (3.7)
African-American	4 (14.8)
Non-Hispanic	27 (100.0)
Education	
Master’s and above	12 (44.4)
Bachelor’s and above	21 (77.8)
Some college	27 (100.0)
Doctorate	5 (18.5)
Prior ED	7 (25.9)
Effective medications taken before prostate cancer (*n* = 9)	6 (66.7)
Time since	
Surgery (months) (*n* = 25), mean ± SD	23.5 ± 11.7
Radiation (months) (*n* = 5), mean ± SD	18.2 ± 13.1
Surgery	25 (92.6)
Robotic	20 (80.0)
Open	5 (20.0)
Nerve-sparring (vs. partial) (*n* = 24)	21 (87.5)
Hormone treatment	4 (14.8)
Surgery followed by radiation	5 (18.5)
Erection hardness score (*n* = 26), mean ± SD	1.48 ± 1.18
Orgasm quality	
Absent	2 (7.4)
Diminished	15 (55.6)
Normal	6 (22.2)
Better	3 (11.1)
Unknown	1 (3.7)
Pain with orgasm (*n* = 25)	0 (0.0)
Urine leakage during sex	
Never	7 (25.9)
Occasionally	14 (51.9)
Always	6 (22.2)
IIEF (*n* = 16), mean ± SD	
Erectile function	16.1 ± 5.8
Orgasmic function	6.6 ± 3.0
Sexual desire	7.3 ± 2.2
Intercourse satisfaction	8.3 ± 3.3
Overall satisfaction	5.5 ± 2.4
SEAR, mean ± SD	
Sexual relationship satisfaction	49.1 ± 26.3
Confidence about self-esteem	66.2 ± 24.8
Overall relationship satisfaction	76.9 ± 24.4

### Theme 1: Importance of education/comprehensive information

The men in our study spoke about the importance of education and comprehensive information before and throughout the process of prostate cancer treatment. Many men who were dissatisfied with their care were upset when they were given misinformation or they felt the information about sexual dysfunction after prostate cancer treatment was not accurate. Although the men who were most unhappy about sexual dysfunction after prostate cancer did not feel well-prepared in terms of their understanding of the impact of prostate cancer treatment on sexual function, the men who had come to terms with their sexual dysfunction felt they had been well-prepared for the sexual side effects of treatment. Men recommended that information be repeated before, during, and after treatment due to challenges in terms of readiness to learn immediately after the cancer diagnosis. They also talked about the challenges of dealing with a stigmatized topic such as sexual dysfunction. The participants said the following:

**Man 21:** “I was not prepared for what was to follow… I think everybody – all the medical staff starting with nursing and support staff and the doctors themselves, they really need to inform the patient with what’s going to happen after the surgery with complications and side effects and on the surgical end and the physical end, but also then that the effects from the surgery should be talked about from the get go so that patients are not surprised, that they know what’s going to be heading their way and if it’s – give them the full information…And I’m very, very emotionally upset about because if I would have known I think I would have been in a better place through the first year and following that first year if I knew.”

**Man 16:** “I was fully informed by everybody. All the doctors that were involved fully informed me that these were things that I was up against if they removed my prostate… So definitely – you definitely must keep everybody informed about what’s going on…It’s really-that’s extremely important ”

### Theme 2: Frustration with sexual dysfunction

The men in our study spoke about their frustration with sexual dysfunction. The men described being upset with the change in their sexual functioning and the impact it had on their intimate relationships. Some men described feelings of loss and grief with changes in orgasm/climax. Three men in the study were pleased to have more intense climax after surgery. The participants said the following:

**Man 12**: “If you have a lack of sensation you don’t have any nocturnal erections. You don’t wake up with an erection and I miss that. I miss it a lot. I miss the sensations of how I used to feel down there, how my body used to feel...I don’t feel whole and I think about it every single day…It’s the first thing I think about in the morning when I wake up and it’s the last thing I think about at night…”

**Man 16:** “I have not – I do not have any recall of having orgasm like I have now. And honest to God there has to be – I mean sometimes I go into mini convulsions because the orgasm lasts and it’s so strong and lasts for probably two minutes.”

### Theme 3: Importance of support and understanding

Not surprisingly, the men and partners we interviewed impressed upon us the need and importance of support and understanding, from their partner and from others in their lives, including their families and support groups like the Us TOO International Prostate Cancer Education and Support Network. An essential element of this support was communication, especially with the partner, which could be very difficult for both parties. To enable this communication, it was crucial for men and their partners to be open about their intimate feelings and concerns. The participants said the following:

**Man 13: “**I’m in a great relationship really for the first time in my life with a woman who really I don’t think doesn’t care what we do as long as we’re together. We enjoy sex a lot not just to be active, just to have intercourse. Without her I don’t think I would be as far along in getting my sex life back to where I want it to be. It’s got to do with her and she’s put up with so much.”

**Partner 1: “**And I think in the beginning he felt we weren’t there for him, the family, because the family – again, the family does think that the man or father or the husband is strong, is – he doesn’t get sick. He’s there, he has the answer, this is how he is. Go, go, go, I’m here, whatever. And then he gets sick and it’s like, okay, you’re done, you’re fine, you had the surgery, it’s okay. But it wasn’t okay to him, but we felt, no, you’re strong, you’re okay. You’re okay. And that kind of wasn’t good because he felt we didn’t care. And we really didn’t understand him.”

**Man 9:** “I come here (prostate cancer support group) shaking like a leaf, man. I get in here with a bunch of guys that had been where I was about to go and man they gassed me up with that strength. And like I said, when I came in I was shaking like a leaf. When I left out I was empowered. “

### Theme 4: Importance of intimacy

Intimacy, both physical and emotional, was a priority for men and their partners. Participants discussed the importance of non-penetrative sex. Most men and their partners felt that non-penetrative sex was a helpful way to maintain intimacy. Some participants felt the relationship was stronger after prostate cancer treatment. The participants said the following:

**Man 21:** “I was fortunate to find this woman and it just enhances every single aspect, whatever, if you’re going to a social event, you’re going on a vacation, you are just being intimate around the house, you’re sharing thoughts and dreams. It just encompasses what life is all about. Some people don’t care about it, but for the men that do it’s devastating.”

**Partner 5:** “Anything I could tell anybody going through this is like, “If you guys are not intimate, and able to talk with each other now you'd better get that straight before the surgery. Better get it straight because you're gonna need each other, and you're gonna need the intimacy more than you've ever had it…”

**Man 15:** “I miss the holding of hands. I miss hugging and things like that. I don’t – that’s not sex in the definition of this survey. But that’s what is available to me in my current physical condition…and so yes, it’s important.”

### Theme 5: Psychological ramifications of sexual dysfunction

Some of the men we interviewed reported psychological issues due to the sexual dysfunction that resulted from prostate cancer treatment. This included many reports of depression and anxiety, in addition to some suicidal ideation. One man reported that he’d rather have his legs cut off than exist in his current state of sexual dysfunction. Men who might not have used clinical terms like “depression” or “anxiety” nevertheless reported the psychologically devastating effects of feeling abnormal, unnatural, and less of a man due to their sexual dysfunction. There was a sense from men and their partners that sexual dysfunction has a great impact on every aspect of life. Like depression, it can change the very lens with which men and their partners view and experience their whole existence. The participants said the following:

**Man 14: “**And that made me very depressed. I was really surprised about that because nowhere in our research prior to my surgery did I run across that a whole lot about how one of the side effects mentally would be depression. And even now I still have some issues with depression, but it’s been over a year and a half and I think I’ve adjusted somewhat because I found that to combat depression I need to stay active, find things that I used to enjoy that I still enjoy and not focus so much on the depression aspect because I had a lot to be pleased about.”

**Man 12: “**The other thing that happened, and again I was not told to expect this, was depression. I had a very serious bout of depression, post op, when I found out the things that were going on with me physically and the time it was taking to get to what I hoped would be healing. I didn’t understand depression. I didn’t know I had it but I suffered with it for several months until I got to the point where I became suicidal.”

### Theme 6: Treatment/provider satisfaction/dissatisfaction

Some men reported dissatisfaction with the treatment they received, while others were happy with the treatment and the care they received around the treatment. The majority of men talked about being happy they were cancer free. Several men talked about their frustration with their provider’s sole focus on the surgery and the lack of resources or help with side effects from treatment. Men who were dissatisfied talked about the lack of support and help from their providers; while men who were satisfied with their care commented on the support and help they got from the provider throughout treatment. Some men felt the provider did not really understand them in terms of their priorities and their feelings about sexual function. Some patients regretted the particular treatment they received or that they received treatment at all. The participants said the following:

**Man 17:** “Uh, we’re alive, ok? I was Gleason 7. My statistical life expectancy would be about 12 years, you know, that’s average. Could be less, could be more. If I did not get treatment. And, uh, my mentality-- and still is—I got it out, out of my body.”

**Man 16:** “I mean, this guy gave us an appointment and sat down with us for two full hours in his office…He sent me home and said do this kind of research and to call him if I had additional questions. Well, after I did a little bit of research, I did have additional questions so I called and he called me back. I mean, I couldn’t believe it. I got a call at home from a doctor. And then he spent another 45 minutes after having spent this two, two and a half hours with me – a half hour after ___ (partner) left and then spent another 40 minutes with me on the telephone. I just thought that was awesome.”

**Man 1:** “I think back. Maybe I shouldn’t have done it (the surgery). And go with the shorter quality of life rather than a long life – a longer life with the situation.”

**Partner 1:** “Because he would always say, maybe I shouldn’t have done that. And I’m like; you did it, so let’s live on and not live in the past…It got better, yeah. He felt like he was just – didn’t want to live because it was – he didn’t feel like a man. He felt like, oh, God, this is a mess.

## Discussion

This study was carried out using an open-ended inquiry into men and their partners’ journeys with sexual dysfunction after prostate cancer treatment. Unlike many qualitative studies that interview with a semi-structured model [7, 14–17] or focus-group model [18], an open-ended question was used to learn the experiences of men and their partners. The majority of the men underwent surgical removal of the prostate (93%).The themes were salient and common across men and partners.

Men emphasized the importance of education and comprehensive information about sexual dysfunction throughout the course of prostate cancer treatment. Men described the need to be better informed about the negative sexual side effects, in order to proactively manage the sexual dysfunction consequences more easily. Yet, this reality was sometimes complicated by the fact that some men were not ready to learn about sexual side effects upon first hearing their cancer diagnosis, as M Ball, et al. [15] found in a study of men post rectal cancer treatment. Nonetheless, similar to findings by M Ball, et al. [15] and N Hanly, S Mireskandari and I Juraskova [7], we found that men insisted that more education at the outset and throughout the process would alleviate anxiety surrounding side effects. The men in our study articulated a clear need for comprehensive information before, during, and after prostate cancer treatment. Men who felt well-prepared and informed reported satisfaction with their care, while the men and partners who did not feel well prepared and informed were unhappy with the care they received.

Some men reported the importance of seeking out information on one’s own, through books, the internet, and support groups such as Us TOO International. They urged their fellow men and partners to do the same. This finding is echoed in KA Krumwiede and N Krumwiede [17] who reported no complaints about a lack of information. Rather, there was a proactive sense of men seeking out information on their own, particularly from men who had already gone through the treatment. This helped men with treatment choices, as it did in our study.

Not surprisingly, another major theme was the distress and frustration caused by sexual dysfunction, which included erectile dysfunction, changes in orgasm and ejaculate, and penile rehabilitation and its accompanying challenges. These frustrations affected both men and their partners and led to a whole range of negative feelings, most notably feeling like less of a man. However, men often assumed their partners were more upset over their sexual dysfunction than they actually were. Few partners reported being upset about their male partners’ sexual dysfunction, although they were aware of the men’s own dissatisfaction. Our study benefited by separately interviewing both men and their partners, as we were able to directly compare what men said to what their partners said. A small minority of men (*n* = 3) reported a more intense climax after surgery, which they found to be a positive experience.

The theme of negative emotions caused by sexual dysfunction is common in the literature [7, 8, 16–19]. In a quantitative study, T Zaider and colleagues [19] showed that regardless of level of sexual function, men who perceive a loss of masculinity following treatment are more likely to be distressed by ED. As most men tied their ED to “feeling like less of a man,” our study confirms this finding. Helping men deal with negative emotions, including grief and loss, is an important part of care.

The psychological ramifications of sexual dysfunction following prostate cancer treatment were articulated by many participants. The majority of men we spoke to reported psychological distress resulting from post-treatment sexual dysfunction, including depression, anxiety, and suicidal ideation. Some sought outside professional counseling or prostate cancer support groups to remedy their situation, and reported positive results in this endeavor. Other studies in the field report similar psychological ramifications as a result of sexual dysfunction [7, 8, 17, 18, 20]. Some men reported the same need to seek outside psychological help, since the primary care provider was not available for such services [7, 20]. N Hanly, S Mireskandari and I Juraskova [7] found not only that the men in their study struggled with depression, but this depression acted as a catalyst for psychological distress due to un-related issues, such as retirement. Offering comprehensive mental health services may be beyond the scope of practice for many urology clinics, yet having a strong referral network of mental health professionals and sexual therapists available will strengthen the care and provide a team-based approach.

The frustration and psychological suffering from sexual dysfunction led men to talk about the importance of support and understanding from others. Others included their partners, their families, and support groups like Us TOO International. Communication was at the heart of this support. Fueling the communication was a sense of openness. Neither communication nor openness was easily achieved. KA Krumwiede and N Krumwiede [17] uncovered the same theme, with a particular emphasis on the gratitude men felt for this support, especially from partners. N Hanly, S Mireskandari and I Juraskova [7], MW Kazer, J Harden, M Burke, MG Sanda, J Hardy and DE Bailey [14], CJ Nelson, S Lacey, J Kenowitz, H Pessin, E Shuk, AJ Roth and JP Mulhall [18] also found this theme. At the heart of this support and understanding from their partners, the men in our study spoke of the importance of intimacy. Romantic relationships are often driven by intimacy, but perhaps our more surprising finding is that many men said that after diagnosis, and especially after treatment, they grew closer to their partners. They were not romanticizing cancer, or wishing it upon others, but some men did say that their relationship to their partner is now better than it was prior to being diagnosed. The process and possibility for physical intimacy was an integral element of this post-treatment emotional intimacy, as men and their partners struggled to resume their sexual lives in the face of men’s post-treatment sexual dysfunction. However, the men reported greater concern with physical intimacy and sex than their partners. To maintain physical intimacy, many couples turned to non-penetrative sex or “outercourse,” such as oral sex, manual stimulation, handholding, and cuddling. N Hanly, S Mireskandari and I Juraskova [7], L Jakobsson, L Persson and P Lundqvist [21] found a similar deepened relationship with spouse. However, many partners in N Hanly, S Mireskandari and I Juraskova [7] were unwilling to engage in outercourse and less supportive with side effects from treatment. Partners that interviewed in our study were positive about outercourse and supportive with side effects from treatment.

Finally, we found a range of experiences with treatment and treatment provider satisfaction. Many men were angry at their providers for being overly optimistic about their post-treatment sexual and urinary functions. Others felt wronged by a lack of care or attention by their surgeons. Some of these men regretted their particular treatment or that they received treatment at all. These findings were echoed in CJ Nelson, S Lacey, J Kenowitz, H Pessin, E Shuk, AJ Roth and JP Mulhall [18]. On the other hand, some men in our study expressed no illusions about the role of the surgeon. The removal of the cancer was the primary job. Other care needs were met with a team based approach, including doctors, nurses, and support staff. Some men expressed deep gratitude towards their doctors and nurses for meeting with them for extended periods of time and answering all their questions and concerns with depth and patience. These patients felt well-informed about all side effects, including sexual side effects. N Hanly, S Mireskandari and I Juraskova [7] also found that men placed trust in doctors who provided ample info and answered lots of questions. MW Kazer, J Harden, M Burke, MG Sanda, J Hardy and DE Bailey [14] reported that men cited their confidence in the healthcare team as one of their reasons for a good recovery. Many men in our study were satisfied with the reality of being cancer-free, with or without sexual dysfunction. Even those men and partners who were frustrated with sexual dysfunction felt grateful to be free of prostate cancer. L Jakobsson, L Persson and P Lundqvist [21] found men expressed a similar gratitude for life, as well as anxiety about death. The health care team plays a crucial role in establishing expectations for care and recovery, and guiding patients and their partners through the journey.

Although this study involved a relatively large group of men and partners from across the country, there were limitations to this study. Men who had been treated for prostate cancer were invited to speak about their experience with sexual dysfunction from the clinic of the principal investigator and from prostate cancer support groups. Thus, men self-selected to participate in the study and their views may not represent the views of other men who did not self-select to participate. Although the sample size is fairly large for a qualitative study, this sample of men was rather homogeneous in race, socio-economic status, and education. The majority of the men in this study underwent surgical removal of the prostate and this is consistent with the most common treatment choice in America. Only 7% of the men in this study had radiation without surgery. Additionally, men were asked post-treatment to describe their pre-treatment sexual function which can lead to recall bias. Future research might include a more diverse sample of men including more men who underwent radiation therapy as a primary treatment. It might also be beneficial to collect information prior to and following treatment.

## Conclusion

This study provides qualitative descriptions of men's and partners' journeys with sex and intimacy after prostate cancer treatment. Prostate cancer survivors and their partners report a need for accurate information about sexual side effects before, during, and after prostate cancer treatment. Men and their partners want providers to be sensitive to their sexuality and assist them in finding appropriate help to deal with sexual dysfunction. Men with sexual dysfunction after prostate cancer treatment report not only frustration with sexual problems, but depression and anxiety. Men identified support and understanding from partners, family, and prostate cancer support groups as an important aspect of the healing process. Involving and engaging partners when disseminating information about intimacy and sex after prostate cancer treatment is also important.

Understanding the impact of the anticipated side effects, like erectile dysfunction, and assisting patients with treating erectile dysfunction, is imperative to treatment satisfaction. Results of this study can be used by healthcare providers to improve care and promote intimacy for the man and his partner by providing comprehensive information about sexual issues throughout and after treatment and providing resources addressing sexual dysfunction and depression and anxiety.

## Abbreviations

ED: Erectile dysfunction
IIEF: International Index of Erectile Function
SEAR: Self-Esteem and Relationship Questionnaire
